# Effects of rumen-protected methionine supplementation on production performance, apparent digestibility, blood parameters, and ruminal fermentation of lactating Holstein dairy cows

**DOI:** 10.3389/fvets.2022.981757

**Published:** 2022-12-12

**Authors:** Yuanxiao Li, Jialin Wei, Mengying Dou, Shuai Liu, Bichuan Yan, Cuiyu Li, Muhammad Zahoor Khan, Yinghui Zhang, Jianxin Xiao

**Affiliations:** ^1^College of Animal Science, Henan University of Science and Technology, Luoyang, China; ^2^Key Laboratory of Low Carbon Culture and Safety Production in Cattle in Sichuan, Animal Nutrition Institute, Sichuan Agricultural University, Chengdu, China; ^3^State Key Laboratory of Animal Nutrition, College of Animal Science and Technology, China Agricultural University, Beijing, China; ^4^Evonik (China) Co., Ltd., Beijing, China; ^5^Department of Animal Science, Faculty of Veterinary and Animal Sciences, University of Agriculture Dera Ismail Khan, Dera Ismail Khan, Pakistan

**Keywords:** dairy cows, rumen-protected methionine, ruminal fermentation, digestibility, blood parameters

## Abstract

This study aimed to evaluate the effects of reducing dietary CP and supplementing rumen protected-methionine (RPM) on production performance, blood parameters, digestibility of nutrients or ruminal fermentation in lactating Holstein dairy cows. A total of 96 lactating cows were randomly assigned to 1 of 2 treatments: a diet containing 17.3% CP without RPM (control group; CON; *n* = 49) or a diet containing 16.4% CP and supplemented with 15.0 g/d of RPM (treatment group; RPM; *n* = 47). No effect was observed in the RPM group on milk yield, milk composition and digestibility of nutrients. The results of blood parameters showed that cows in the RPM group exhibited lower blood urea nitrogen concentration than in CON group. Rumen microbial crude protein (MCP) was higher in the RPM group compared to the CON group. Ruminal volatile fatty acid (VFA) concentrations were not different between treatments except for butyrate and isovalerate, which were higher in the RPM group than the CON group 2 h after feeding. In conclusion, reducing dietary CP with RPM supplementation did not limit milk yield, milk composition or digestibility of nutrients, but could improve nitrogen utilization, synthesis of MCP and partially increase VFA production 2 h after feeding cows.

## Introduction

In order to satisfy their protein requirements, cows might be fed excessive dietary crude protein (CP). Most of this CP ends up being excreted as urea, which contribute to the atmospheric N through NO_2_ emissions (1) or be hydrolyzed to NH_3_ and volatilized (2), ultimately harming the environment. In addition, some researchers have reported the negative impact of high dietary CP on N utilization in dairy cows (3). Moreover, milk production and composition were not improved when dietary CP was increased to 18 (2) or 18.8% (4). Meanwhile, dietary protein makes up a large proportion of feeding costs, about 40% (5), such that feeding excess protein might reduce the farm's profit margins. In consideration of the factors mentioned above, decreasing dietary CP has attracted great interest from researchers across the world. Besides the level of dietary CP, previous studies have shown that ensuring ideal AA balance, especially methionine (Met) and lysine (Lys) (2), is key to formulating rations that contribute to reduced N excretion (6) while adequately supporting productive performance.

Given that rumen undegradable protein (RUP) is important in meeting the daily protein needs of cows, especially during the lactation stage, alternative strategies of feeding rumen-protected AA need to be considered. More studies have explored the effects of feeding rumen-protected methionine (RPM) in dairy cows in recent years. Several studies indicated that reducing the dietary CP diet and supplementing RPM could increase milk, protein, fat, and lactose yields (7–9). The increased yields could be associated with higher dry matter intake (DMI) reported in lactating cows fed RPM (10). In addition, RPM supplemention may allow the reduced CP diets to improve N efficiency and reduce urinary N excretion (11, 12) and methane gas (13). Although, the role of reduced dietary CP levels with RPM supplementation on milk production performance, N efficiency and environmental impact has been fairly discussed, other aspects such as nutrient digestibility, rumen health and function need to be explored further.

Thus, the objective of the present study was to investigate the effects of reducing dietary CP levels and supplementing RPM on production performance, apparent digestibility of nutrients, blood parameters, and ruminal fermentation in lactating Holstein dairy cows. We hypothesized that reducing dietary CP levels and supplementing RPM would not limit milk yield and composition, but could change the rumen fermentation profile, increase nutrient digestibility, and alter blood parameters.

## Materials and methods

### Treatments, experimental diet, and cows management

This study was conducted at Shengsheng Dairy Farm in Luoyang City, Henan Province, China. Animal management and experimental procedures were approved by the Animal Care Committee of China Agricultural University. A total of 96 lactating Holstein dairy cows (63 ± 25 d in milk; 34.4 ± 5.74 kg/d of milk production; mean ± SD) were randomly assigned to 1 of 2 dietary treatments and housed in two separate free-stall barns. The experiment lasted 75 d and consisted of 15 d for diet adaptation and 60 d for data collection. Diets were fed as total mixed ration (TMR; Table 1): diet containing 17.3% CP without RPM (control group; CON; *n* = 49) or diet containing 16.4% CP and supplemented with 15.0 g/d of RPM (treatment group; RPM; *n* = 47). The CON diet had a Lys:Met ratio of 3.39:1, while the RPM diet was 2.84:1. The RPM used in the study was Mepron^®^ provided by Evonik (China) Co., Ltd. Mepron^®^ is produced by coating methionine with a protective film. The TMR was fed three times daily at 0800, 1,200, and 1,700 h. Cows had free access to feed and water. The amount of feed offered daily was adjusted to achieve a 3–5% orts.

**Table 1:** Ingredients and nutrient composition in diets^a^.

**Item**	**CON**	**RPM**
**Ingredients (% DM)**	
Alfalfa hay	11.3	11.3
Oat hay	3.7	3.7
Corn silage	45.2	44.9
Whole cottonseed	2.5	5.0
Beet pulp	2.5	2.5
Molasses	3.5	3.5
Fat power	0.5	0.5
Sodium bicarbonate	0.4	0.4
Yeast	0.5	0.5
Steam flaked corn	1.3	3.7
Ground corn grain	12.1	10.0
Wheat bran	2.0	2.0
Corn gluten feed	1.5	1.5
Distillers dried grains with solubles	1.5	1.5
Vitamins and trace minerals	1.6	1.6
Whole soybean	0.8	0.8
Soybean meal	9.0	6.2
Expeller-pressed cottonseed	—	0.5
Mepron^®^^b^	—	0.04
**Nutrient composition** ^**c**^	
DM (%)	61.4	61.6
NE${\text{NE}}_{\text{L}}^{\text{d}}$ (M.cal/kg)	1.7	1.7
CP (% DM)	17.3	16.4
RDP (% DM)	11.7	11.0
NDF (% DM)	31.3	32.3
ADF (% DM)	18.4	19.0
peNDF (% DM)	23.3	24.6
NFC^e^ (% DM)	35.5	36.0
Fat (% DM)	6.0	6.0
Met (% MP)	1.92	2.27
Lys (% MP)	6.51	6.45
Lys: Met ratio	3.39:1	2.84:1

a Treatments CON and RPM groups consisted of a total mixed ration containing 17.3 and 16.4% CP, respectively.

b Rumen-protected Met product from Evonik (China) Co., Ltd. (DL-Methionine min. % 85.0).

c DM, Dry matter; NEL, net energy for lactation; CP, crude protein; NDF, neutral detergent fiber; ADF, acid detergent fiber; peNDF, physically effective neutral detergent fiber; NFC, non-fiber carbohydrate; Met, methionine; Lys, lysine.

^d^ Estimated using the NRC (14) model based on DM intakes.

e NFC = 100 (%NDF–NDIN × 6.25) – % CP – % fat – % ash.

### Sample collection and analysis

Cows were milked 3 times per day for the duration of the experiment. Milk samples were collected from each cow 3 times per day at 0700, 1,100, and 1,600 h and mixed well in a 4:3:3 ratio as one sample, then analyzed for fat, true protein, lactose, milk urea nitrogen (MUN), somatic cell count (SCC), solids-not-fat (SNF), total solid (TS) and freezing point (FP) at the Luoyang Dairy Cow Center (Luoyang, China). The TMR samples were collected 3 times per day during the experimental period and mixed well to determine the feed composition. Samples were dried at 65°C for 48 h until a constant weight was obtained. Then, dried TMR samples were ground through a 1-mm screen (KRT-34; KunJie, Beijing, China) and analyzed for DM using method 950.15 of Association of Official Analytical Chemists (15). Nitrogen was analyzed by the method 984.13 (15) and crude protein (CP) was measured by multiplying 6.25 by the nitrogen content. Neutral detergent fiber (NDF) and acid detergent fiber (ADF) were measured according to the method described by Van Soest et al. (16). Ash (method 942.05) was measured according to the AOAC (17).

Fifteen Holstein dairy cows per group were randomly selected with similar milk yield and days in milk to collect blood, fecal grab and rumen fluid samples. On d 0, 30 and 60 of the experimental periods, blood samples were collected from the coccygeal vein into 10 mL heparin-coated tubes (Vacutainer; Becton Dickinson, Franklin Lakes, NanJing, China) before morning feeding. Blood samples were immediately centrifuged at 3,500 × g at 4°C for 15 min to obtain plasma samples and stored at −20°C for further analysis. Concentrations of total protein (TP), albumin (ALB), globulin (GLB), blood urea nitrogen (BUN), cholesterol (CHO), aspartate transaminase (AST), alanine transaminase (ALT), and alkaline phosphatase (ALP) were measured at a commercial laboratory (People's Liberation Army 534 Hospital, China).

Fecal grab samples were collected 4 times (0300, 0800, 1,300, and 1,800 h) each day on d 58, 59, and 60 from these selected cows. The samples of each cow were evenly mixed to make a simple composite and taken to about 200 g, adding 10% tartaric acid of 1/4 fecal weight to fix nitrogen. These fecal grab samples were held at 65°C in a forced-air oven until completely dried and were ground to pass a 1-mm screen (KRT-34; KunJie, Beijing, China). Fecal samples were analyzed for DM, NDF, ADF, CP, and ash according to the AOAC as described earlier. The acid-insoluble ash (AIA) was used as an internal marker to determine the apparent digestibility of nutrients. TMR, orts and fecal samples were analyzed according to the procedures by Vankeulen and Young (18). The apparent digestibility of nutrients was calculated as follows:


$$\begin{array}{l} \text{Apparent Digestibility of Nutrients}\\ =\left[1-\left(\text{Ad}\times \text{Nf}\right)/\left(\text{Af}\times \text{Nd}\right)\right]\times 100. \end{array}$$

where Ad = AIA in the diets (g/kg); Af = AIA in the feces (g/kg); Nd = the concentration of a nutrient in the diet (g/kg); Nf = the concentration of a nutrient in the feces (g/kg).

On the last day of the experiment, rumen fluid samples were collected from the selected 15 cows per group. Samples were collected before morning feeding and 2, 4, 6, 8, 12 h after morning feeding by a 200 mL esophageal tube. Rumen fluid was filtered through four layers of gauze, centrifuged at 1,800 × g for 15 min and 1 mL of the supernatant collected and acidified with 4.5 mL of 0.2 mol/L HCL for later analysis of ammonium nitrogen. Meanwhile, l mL 25% metaphosphate acid was added to 4 mL of the supernatant and stored at −20°C for later analysis of microbial crude protein (MCP), ammonia N (NH_3_-N) (analyzing in the Evonik (China) laboratory, Beijing) and volatile fatty acids (VFA), including acetate, propionate, butyrate, valerate, isobutyrate, and isovalerate (using Shimadzu GC-7A gas chromatograph, Japan).

### Statistical analysis

All data were analyzed using SAS (SAS version 9.2, SAS Institute Inc., Cary, NC, USA) with cow as the experimental unit. Milk yield, milk composition, apparent digestibility of nutrients, blood parameters, and rumen fermentation products were analyzed using the PROC MIXED procedure of SAS. Milk yield, milk composition and blood parameters were calculated by averaging the samples collected in d 0, d 30 and d 60 and then analyzing. The model of rumen fermentation products included the fixed effects of treatment, time, and time × treatment interaction, and cow within treatment as a random effect. Degrees of freedom were counted using the Kenward-Roger approximation option of the MIXED procedure. In order to explicate the repeated measures within-subject, the covariance structures were executed for each repeated variable based on the best fit determined by the Akaike information criterion. A significant difference between the treatment and control group was declared at p < 0.05 and tendencies were considered when 0.05 ≤ *p* < 0.10. A highly significant difference was indicated at *p* ≤ 0.01.

## Results and discussion

### Milk yield and composition

The results of the milk yield and composition are shown in Table 2. Milk yield, fat, protein, lactose, TS, MUN, SCC, and FP were similar between the CON and RPM groups. There was a trend for lower SNF with decreased CP feeding and RPM supplementation (*p* = 0.09). Our findings implied that decreasing dietary CP to 16.4% with RPM supplementation and a relatively constant fermentable carbohydrate failed to affect the milk yield and composition negatively. The results are consistent with other trials that found milk production performance was similar when dietary AA was well balanced, and differences in CP of the diets ranged between 13 and 18.6% (2, 9, 11). Moreover, several trials have demonstrated that RPM supplementation increased milk (7, 19) and milk protein (20) yields. In contrast, it has been reported that milk yield or milk composition, including fat, lactose and SNF, were significantly decreased when cows were fed 11% CP as opposed to 13, 15, and 17% CP (9). The diets of 11, 13, 15, and 17% CP were supplemented with RPM, which provided 4.2, 8.1, 10.3, and 12.4 g/d of absorbed Met respectively.

**Table 2:** Effects of supplementing with rumen-protected methionine (RPM) on milk yield, milk composition.

**Item^b^**	**Treatment** ^**a**^		**SEM**	***p-*value**
	**CON**	**RPM**		
Milk yield, kg/day	36.8	36.8	0.07	1.00
FP (°F)	31.0	31.1	0.94	0.40
SCC (cell/mL)	153,100	115,900	0.42	0.38
**Milk composition, %**				
Fat	3.49	3.80	0.03	0.24
Protein	3.39	3.34	0.004	0.36
Lactose	4.85	4.87	0.01	0.82
TS	12.01	11.97	0.04	0.32
SNF	8.59	8.41	0.01	0.09
MUN (mg/dL)	15.44	14.82	0.07	0.35

a Treatments CON and RPM groups consisted of a total mixed ration containing 17.3 and 16.4% CP, respectively.

b FP, Freezing point; SCC, somatic cell count; TS, total solid; SNF, solids-not-fat; MUN: milk urea nitrogen.

Although certain ranges of difference in CP might not influence milk yield, several studies have highlighted the high sensitivity of SNF to dietary CP content. As has been shown by researchers who fed cows tat 17.3 or 16.1% CP (11), reducing dietary CP might significantly reduce the milk SNF yield, which is a key factor that could affect milk payment. Results from the current study differed from the report of Broderick et al. (11) because cows were predicted to be in negative balance in their study, whether energy or mean N. However, in the current study, diets were nutritionally balanced in consideration of normal milk yield and DMI.

The role of EAA in MUN has been discussed widely. MUN is a key indicator that can estimate whether a diet provides excessive protein (21). A previous study found that MUN concentration was lower in cows fed supplementing rumen-protected essential amino acids while reducing dietary protein (22). Compared with feeding an 18% CP diet, MUN values decreased in lactating cows fed either 16.4 or 15.6% CP diets supplemented with RPM (2). An earlier study reported no difference in MUN concentration in cows fed diets that differed by 1% in dietary CP content (23). The similar content in MUN in our study could be explained by the small differences (0.9%) in dietary CP between the two experimental groups. The SCC level, an indicator of mammary health, often increases when infection occurs in the mammary gland (24, 25). The SCC values were within the normal ranges (100,000–314,000 cells/mL) (26) between the two groups.

### Blood parameters

The effects of supplementing RPM on blood parameters are shown in Table 3. The BUN concentration was lower (*p* < 0.001) in RPM cows compared to the CON group. Other blood parameters, such as ALT, AST, TP, ALB, GLB, CHO and ALP, did not exhibit any differences between groups. This result was not anticipated, but it is in agreement with published data (2).

**Table 3:** Effects of supplementing with rumen-protected methionine (RPM) on blood parameters.

**Item^b^**	**Treatment** ^**a**^		**SEM**	***p-*value**
	**CON**	**RPM**		
ALT (U/L)	26.62	27.00	0.25	0.88
AST (U/L)	88.38	99.00	0.99	0.28
TP (g/L)	73.74	74.20	0.14	0.75
ALB (g/L)	28.55	28.06	0.09	0.59
GLB (g/L)	45.19	46.13	0.18	0.61
ALB: GLB ratio	0.64	0.62	0.004	0.49
BUN (mmol/L)	5.30	4.14	0.03	< 0.001
CHO (mmol/L)	5.65	5.16	0.05	0.30
ALP (U/L)	56.69	53.58	0.61	0.63

a Treatments CON and RPM groups consisted of total mixed ration containing 17.3 and 16.4% CP, respectively.

b TP, Total protein; ALB, albumin; GLB, globulin; BUN, blood urea nitrogen; CHO, cholesterol; AST, aspartate transaminase; ALT, alanine transaminase; ALP, alkaline phosphatase.

The decline in BUN in our study accords with a previous study in which cows were fed a low-protein diet supplemented with RPM while reducing protein level (2, 27). Lower dietary CP and rumen degradable protein (RDP) are most likely to reduce the BUN concentrations in lactating dairy cows (28). However, reducing dietary protein should be accompanied by balancing dietary AA, especially the Lys to Met ratio, to enhance protein utilization in dairy cows (22), as we did in the current study by supplementing RPM in the diets. Low BUN has been associated with improved protein utilization (29), and reduced BUN concentrations could stimulate rumen urea transfer and increase urea transport rates (30). In addition, some previous studies reported that reduced BUN and MUN could promote reproductive performance in dairy cows (31, 32).

### Apparent digestibility of nutrients

The results of the apparent digestibility of nutrients in dairy cows are shown in Table 4. The total apparent digestibility of DM, CP, NDF, ADF and ash were not affected by the treatment, despite the difference in CP levels.

**Table 4:** Effects of supplementing with rumen-protected methionine (RPM) on the apparent digestibility of nutrients.

**Item^b^ (%)**	**Treatment** ^**a**^		**SEM**	***p-*value**
	**CON**	**RPM**		
DM	65.09	64.44	0.32	0.83
CP	71.43	72.65	0.30	0.70
NDF	48.60	41.35	0.51	0.13
ADF	38.88	44.59	0.69	0.38
Ash	53.43	55.17	0.34	0.59

a Treatments CON group and RPM group consists of a total mixed ration containing 17.3 and 16.4% CP, respectively.

b DM, Dry matter; CP, crude protein; NDF, neutral detergent fiber; ADF, acid detergent fiber.

Feed digestibilities of OM, NDF, and ADF have been associated with dietary CP content (11). Low CP (<15% of DM) and RDP (<10% of DM) concentrations can reduce NDF digestibility (11, 33). Earlier studies showed that a diet with 9% RDP lowered OM digestibility (34). Inadequate RDP could have a negative effect on utilizing NH_3_-N, then low NH_3_-N efficiency could reduce fiber digestion and microbial growth (35). However, control and RPM diets used in our research might have provided adequate RDP, 11.7 and 11%, respectively, resulting in no impact on nutrient digestibility. Our results are consistent with a previous study which showed that the apparent digestibility of DM, OM, NDF, CP and starch were not different whether or not RPM was added and RDP was adequate (10% of DM) (33). In line with the above-mentioned results, more urea was recycled to the rumen when dietary CP was higher than 16%, and ruminal NH_3_-N concentrations in Table 5 were above the minimum requirement (≥ 5 mg/dL) of ruminal NH_3_-N concentration for rumen microbial growth (36) to further support the findings of this study. Although a large proportion of protected methionine escaped ruminal degradation, a small fraction of methionine was still released into the rumen. Salsbury et al. (37) observed that unprotected supplemental methionine enhanced ruminal bacteria growth rate *in vitro*. So, supplemental rumen-protected methionine may change the community composition of the rumen microbiota and their metabolism, and feed digestibility will be expected to increase with the activity increasing of rumen bacteria. However, our results showed no difference on feed digestibility between RPM and CON group. They indicated that a small fraction of methionine released from the rumen-protected supplement may not have affected the growth of major bacterial species in the rumen (10). No effect on nutrient digestibility by supplemented RPM was observed when diet CP was higher than 16%.

**Table 5:** Effects of supplementing with rumen-protected methionine (RPM) on the Rumen Fermentation Products of dairy cows.

**Items^b^**	**Treatment** ^**a**^		**SEM^c^**	***p***		
	**CON**	**RPM**		**Treatment**	**Time**	**Treatment × Time**
MCP	116	135	5.33	0.01	<0.001	0.56
Ammonia nitrogen (mg/dL)	7.85	9.60	0.49	0.15	0.001	0.24
**VFA (mmol/L)**						
Acetate	38.55	40.48	1.67	0.62	0.002	0.11
Propionate	19.38	19.99	1.22	0.78	<0.001	0.09
Butyrate	9.25	10.00	0.43	0.37	<0.001	0.03
Valerate	1.54	1.45	0.07	0.52	0.018	0.10
Isobutyrate	1.03	1.06	0.07	0.82	<0.001	0.74
Isovalerate	1.84	1.66	0.09	0.28	<0.001	<0.001
Acetate:propionate ratio	2.55	2.79	0.21	0.63	0.007	0.99

a Treatments CON and RPM groups consisted of a total mixed ration containing 17.3 and 16.4% CP, respectively.

b MCP, Microbial crude protein; VFA, volatile fatty acid.

c Standard error of Means.

### Rumen fermentation

**Figure 1:**
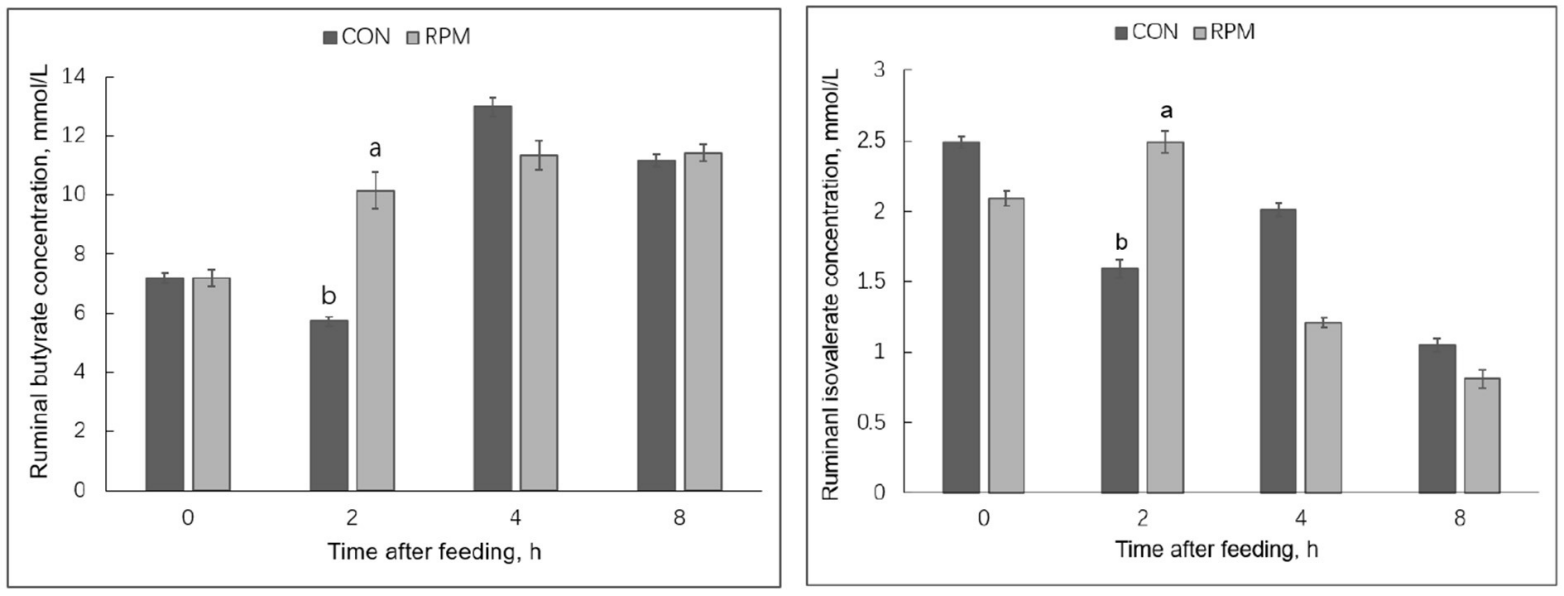
Ruminal butyrate and isovalerate concentrations in Holstein cows fed a control diet 1 (CON) containing 17.3% CP or treatment diet containing 16.4% CP with RPM supplementation (RPM) at 0, 2, 4, and 8 h after feeding. ^a,b^Least squares means within a row with different superscripts differ (*p* < 0.05). Bars indicate the standard error of the means.

Effects of experimental diets on rumen fermentation products are shown in Table 5. The results showed that MCP in the RPM group was higher than in the CON group (*p* = 0.006). All rumen fermentation products were significantly affected by time (*p* < 0.05), but no differences were found between treatments. Treatment × time interaction effects (*p* < 0.05) were observed for ruminal butyrate and isovalerate concentrations, with cows fed RPM having higher butyrate and isovalerate concentrations 2 h after feeding, as shown in Figure 1. The mean concentration of NH_3_-N was numerically higher in the RPM diet; however, the effect was not statistically significant.

Unprotected methionine has been demonstrated to promote rumen bacterial growth and protein synthesis *in vitro* (37, 38). Although we are unsure if rumen bacterial growth rates were altered in our study, we did observe a higher ruminal MCP concentration in cows fed RPM with reduced dietary CP. Similar results were also observed in a study by other researchers, who found that MCP content could be increased *in vitro* by supplementing RPM (0.81 g/kg DM) in a low-protein diet (13).

Although no effects were found in VFA concentration during the whole experimental period, cows fed RPM had higher ruminal butyrate and isovalerate concentration 2 h after feeding, consistent with a previous animal study (39) and an *in vitro* study (13). Ruminal VFA production is dependent on the type of feed substrate, microbial composition, and extent of fiber degradation (40). Using a rumen simulation technique, Abbasi et al. (13) incubated rumen inoculum under high or low dietary CP conditions with or without RPM. These authors found that the abundance of *Ruminococcus albus* was highest under low dietary protein with high RPM. Moreover, an *in sacco* study utilizing methionine analogs [2-hydroxy-4-(methylthio) butanoic acid (HMB) and its isopropyl ester (HMBi)] reported higher VFA concentrations and ruminal abundance of *F. succinogenes* and *R. flavefaciens*, two important cellulolytic bacteria (41). The increase in ruminal butyrate and isovalerate concentration at 2 h after feeding might imply that the RPM positively affected the microbiota that ferment these VFAs. Future studies should include rumen microbial analysis to confirm these findings in animal studies.

## Conclusion

Our results demonstrated that milk yield and milk composition were not negatively impacted by the dietary CP composition. Furthermore, feeding 16.4% CP with RPM could improve nitrogen utilization, synthesis of MCP and partially increase VFA production 2 h after feeding dairy cows. More research is required to explore the mechanisms by which RPM supplementation improves rumen function in lactating dairy cows.

## Data availability statement

The raw data supporting the conclusions of this article will be made available by the authors, without undue reservation.

## Ethics statement

The animal study was reviewed and approved by this study was conducted at Shengsheng Dairy Farm in Luoyang City, Henan Province, China. Animal management and experimental procedures were approved by the Animal Care Committee of China Agricultural University.

## Author contributions

YL and YZ: conceived the idea and designed the experiments. YL and JX: implemented the experiment. JW, MK, BY, MD, and CL: performed the analysis. JW and SL: wrote the manuscript. All authors read and approved the final manuscript.

## References

[1] Marshall CJ Beck MR Garrett K Barrell GK Al-Marashdeh O Gregorini P. Grazing dairy cows with low milk urea nitrogen breeding values excrete less urinary urea nitrogen. Sci Total Environ. (2020) 739:139994. 10.1016/j.scitotenv.2020.139994 32535469

[2] Bahrami-Yekdangi H Khorvash M Ghorbani GR Alikhani M Jahanian R Kamalian E. Effects of decreasing metabolizable protein and rumen-undegradable protein on milk production and composition and blood metabolites of Holstein dairy cows in early lactation. J Dairy Sci. (2014) 97:3707–14. 10.3168/jds.2013-6725 24679928

[3] Huhtanen P Hristov AN. A meta-analysis of the effects of dietary protein concentration and degradability on milk protein yield and milk N efficiency in dairy cows. J Dairy Sci. (2009) 92:3222–32. 10.3168/jds.2008-1352 19528599

[4] Leonardi C Stevenson M Armentano LE. Effect of two levels of crude protein and methionine supplementation on performance of dairy cows. J Dairy Sci. (2003) 86:4033–42. 10.3168/jds.S0022-0302(03)74014-4 14740841

[5] St-Pierre NR. The Costs of Nutrients, Comparison of Feedstuffs Prices the Current Dairy Situation. (2012). Available online at: https://dairy.osu.edu/newsletter/buckeye-dairy-news/volume-13-issue-6/costs-nutrients-comparison-feedstuffs-prices-and.

[6] Broderick GA. Effects of varying dietary protein and energy levels on the production of lactating dairy cows. J Dairy Sci. (2003) 86:1370–81. 10.3168/jds.S0022-0302(03)73721-7 12741562

[7] Schmidt J Sipócz P Cenkvári E Sipócz J. Use of protected methionine (Mepron M 85) in cattle. Acta Vet Hung. (1999) 47:409–18. 10.1556/avet.47.1999.4.2 10641332

[8] Berthiaume R Thivierge MC Patton RA Dubreuil P Stevenson M McBride BW. Effect of ruminally protected methionine on splanchnic metabolism of amino acids in lactating dairy cows. J Dairy Sci. (2006) 89:1621–34. 10.3168/jds.S0022-0302(06)72229-9 16606732

[9] Nursoy H Ronquillo MG Faciola AP Broderick G. Lactation response to soybean meal and rumen-protected methionine supplementation of corn silage-based diets. J Dairy Sci. (2018) 101:2084–95. 10.3168/jds.2017-13227 29290449

[10] Abdelmegeid MK Elolimy AA Zhou Z Lopreiato V McCann J Loor JJ. Rumen-protected methionine during the peripartal period in dairy cows and its effects on abundance of major species of ruminal bacteria. J Animal Sci Biotechnol. (2018) 9:17. 10.1186/s40104-018-0230-8 29445454 PMC5801671

[11] Broderick GA Stevenson MJ Patton RA Lobos N Olmos Colmenero JJ. Effect of supplementing rumen-protected methionine on production and nitrogen excretion in lactating dairy cows. J Dairy Sci. (2008) 91:1092–102. 10.3168/jds.2007-0769 18292265

[12] Zhao Y Rahman MS Zhao G Bao Y Zhou K. Dietary supplementation of rumen-protected methionine decreases the nitrous oxide emissions of urine of beef cattle through decreasing urinary excretions of nitrogen and urea. J Sci Food Agric. (2020) 100:1797–805. 10.1002/jsfa.10217 31849061

[13] Abbasi IHR Abbasi F Liu L Bodinga BM Abdel-Latif M Swelum AA. Rumen-protected methionine a feed supplement to low dietary protein: effects on microbial population, gases production and fermentation characteristics. AMB Exp. (2019) 9:93. 10.1186/s13568-019-0815-4 31243611 PMC6595026

[14] National Research Council. (2001). Nutrient Requirements of Dairy Cattle. 6th Rev. Ed. National Academy of Sciences, Washington, D.C.

[15] AOAC. Official Methods of Analysis, 15th Edn. Arlington, VA: Association of Official Analytical Chemists (1990).

[16] Van Soest PJ Robertson JB Lewis BA. Methods for dietary fiber, neutral detergent fiber, and nonstarch polysaccharides in relation to animal nutrition. J Dairy Sci. (1991) 74:3583–97. 10.3168/jds.S0022-0302(91)78551-2 1660498

[17] AOAC. Official Methods of Analysis. 17th Edn. Gaithersburg, MD: Association of Official Analytical Chemists (2000).

[18] Vankeulen J Young BA. Evaluation of acid-insoluble ash as a natural marker in ruminant digestibility studies. J Anim Sci. (1977) 44:282–7. 10.2527/jas1977.442282x

[19] Patton RA. Effect of rumen-protected methionine on feed intake, milk production, true milk protein concentration, and true milk protein yield, and the factors that influence these effects: a meta-analysis. J Dairy Sci. (2010) 93:2105–18. 10.3168/jds.2009-2693 20412926

[20] Zanton GI Bowman GR Vázquez-Añón M Rode LM. Meta-analysis of lactation performance in dairy cows receiving supplemental dietary methionine sources or postruminal infusion of methionine. J Dairy Sci. (2014) 97:7085–101. 10.3168/jds.2014-8220 25242429

[21] Johnson RG Young AJ. The association between milk urea nitrogen and DHI production variables in western commercial dairy herds. J Dairy Sci. (2003) 86:3008–15. 10.3168/jds.S0022-0302(03)73899-5 14507038

[22] Arriola Apelo SI Bell AL Estes K Ropelewski J de Veth M Hanigan MD. Effects of reduced dietary protein and supplemental rumen-protected essential amino acids on the nitrogen efficiency of dairy cows. J Dairy Sci. (2014) 97:5688–99. 10.3168/jds.2013-7833 25022689

[23] Flis SA Wattiaux MA. Effects of parity and supply of rumen-degraded and undegraded protein on production and nitrogen balance in Holsteins. J Dairy Sci. (2005) 88:2096–106. 10.3168/jds.S0022-0302(05)72886-1 15905440

[24] Le Roux Y Laurent F Moussaoui F. Polymorphonuclear proteolytic activity and milk composition change. Vet Res. (2003) 34:629–45. 10.1051/vetres:2003021 14556698

[25] Hernández-Ramos PA Vivar-Quintana AM Revilla I. Estimation of somatic cell count levels of hard cheeses using physicochemical composition and artificial neural networks. J Dairy Sci. (2019) 102:1014–24. 10.3168/jds.2018-14787 30591330

[26] Nasr MAF El-Tarabany MS. Impact of three THI levels on somatic cell count, milk yield and composition of multiparous Holstein cows in a subtropical region. J Therm Biol. (2017) 64:73–7. 10.1016/j.jtherbio.2017.01.004 28166949

[27] Sun F Cao Y Cai C Li S Yu C Yao J. Regulation of nutritional metabolism in transition dairy cows: energy homeostasis and health in response to post-ruminal choline and methionine. PLoS ONE. (2016) 11:e0160659. 10.1371/journal.pone.0160659 27501393 PMC4976856

[28] Bahrami-Yekdangi M Ghorbani GR Khorvash M Khan MA Ghaffari MH. Reducing crude protein and rumen degradable protein with a constant concentration of rumen undegradable protein in the diet of dairy cows: production performance, nutrient digestibility, nitrogen efficiency, and blood metabolites. J Anim Sci. (2016) 94:718–25. 10.2527/jas.2015-9947 27065142

[29] Bottini-Luzardo MB Aguilar-Perez CF Centurion-Castro FG Solorio-Sanchez FJ Ku-Vera JC. Milk yield and blood urea nitrogen in crossbred cows grazing Leucaena leucocephala in a silvopastoral system in the Mexican tropics. Trop Grasslands-Forrajes Tropicales. (2016) 4:159–67. 10.17138/TGFT(4)159-167

[30] Muscher AS Schröder B Breves G Huber K. Dietary nitrogen reduction enhances urea transport across goat rumen epithelium. J Anim Sci. (2010) 88:3390–8. 10.2527/jas.2010-2949 20581287

[31] Butler WR Calaman JJ Beam SW. Plasma and milk urea nitrogen in relation to pregnancy rate in lactating dairy cattle. J Anim Sci. (1996) 74:858–65. 10.2527/1996.744858x 8728008

[32] Ramirez-Valverde R Misztal I Bertrand JK. Comparison of threshold vs linear and animal vs sire models for predicting direct and maternal genetic effects on calving difficulty in beef cattle. J Anim Sci. (2001) 79:333–8. 10.2527/2001.792333x 11219441

[33] Lee C Giallongo F Hristov AN Lapierre H Cassidy T Heyler KS. Effect of dietary protein level and rumen-protected amino acid supplementation on amino acid utilization for milk protein in lactating dairy cows. J Dairy Sci. (2015) 98:1885–902. 10.3168/jds.2014-8496 25547302

[34] Stokes SR Hoover WH Miller TK Blauweikel R. Ruminal digestion and microbial utilization of diets varying in type of carbohydrate and protein. J Dairy Sci. (1991) 74:871–81. 10.3168/jds.S0022-0302(91)78236-2 1712798

[35] Allen MS. Effects of diet on short-term regulation of feed intake by lactating dairy cattle. J Dairy Sci. (2000) 83:1598–624. 10.3168/jds.S0022-0302(00)75030-2 10908065

[36] Satter LD Roffler RE. Nitrogen requirement and utilization in dairy cattle. J Dairy Sci. (1975) 58:1219–37. 10.3168/jds.S0022-0302(75)84698-4 1099126

[37] Salsbury RL Marvil DK Woodmansee CW Haenlein G. Utilization of methionine and methionine hydroxy analog by rumen microorganisms *in vitro*. J Dairy Sci. (1971) 54:390–6. 10.3168/jds.S0022-0302(71)85850-2 5106928

[38] Gil LA Shirley RL Moore JE. Effect of methionine hydroxy analog on bacterial protein synthesis from urea and glucose, starch or cellulose by rumen microbes, *in vitro*. J Anim Sci. (1973) 37:159–63. 10.2527/jas1973.371159x 4737210

[39] Noftsger S St-Pierre NR Sylvester JT. Determination of rumen degradability and ruminal effects of three sources of methionine in lactating cows. J Dairy Sci. (2005) 88:223–37. 10.3168/jds.S0022-0302(05)72680-1 15591385

[40] Bannink A Kogut J Dijkstra J France J Kebreab E Van Vuuren A. Estimation of the stoichiometry of volatile fatty acid production in the rumen of lactating cows. J Theor Biol. (2006) 238:36–51. 10.1016/j.jtbi.2005.05.026 16111711

[41] Martin C Mirande C Morgavi DP Forano E Devillard E. Methionine analogues HMB and HMBi increase the abundance of cellulolytic bacterial representatives in the rumen of cattle with no direct effects on fibre degradation. Animal Feed Sci Tech. (2013) 182:16–24. 10.1016/j.anifeedsci.2013.03.008

